# Molecular phylogeny of the megadiverse insect infraorder Bibionomorpha *sensu lato* (Diptera)

**DOI:** 10.7717/peerj.2563

**Published:** 2016-10-18

**Authors:** Jan Ševčík, David Kaspřák, Michal Mantič, Scott Fitzgerald, Tereza Ševčíková, Andrea Tóthová, Mathias Jaschhof

**Affiliations:** 1 Faculty of Science, Department of Biology and Ecology, University of Ostrava, Ostrava, Czech Republic; 2 Pacific Northwest Diptera Research Lab, Corvallis, OR, United States of America; 3 Faculty of Science, Department of Botany and Zoology, Masaryk University, Brno, Czech Republic; 4 Station Linné, Färjestaden, Sweden

**Keywords:** Lower Diptera, Sciaroidea, Phylogenetic analysis, Molecular markers, Systematics

## Abstract

The phylogeny of the insect infraorder Bibionomorpha (Diptera) is reconstructed based on the combined analysis of three nuclear (18S, 28S, CAD) and three mitochondrial (12S, 16S, COI) gene markers. All the analyses strongly support the monophyly of Bibionomorpha in both the narrow (*sensu stricto*) and the broader (*sensu lato*) concepts. The major lineages of Bibionomorpha * sensu lato* (Sciaroidea, Bibionoidea, Anisopodoidea, and Scatopsoidea) and most of the included families are supported as monophyletic groups. Axymyiidae was not found to be part of Bibionomorpha nor was it found to be its sister group. Bibionidae was paraphyletic with respect to Hesperinidae and Keroplatidae was paraphyletic with respect to Lygistorrhinidae. The included Sciaroidea *incertae sedis* (except * Ohakunea* Edwards) were found to belong to one clade, but the relationships within this group and its position within Sciaroidea require further study.

## Introduction

Bibionomorpha is one of the approximately 10 infraorders currently recognized in the megadiverse insect order Diptera (Wood & Borkent, 1989; Oosterbroek & Courtney, 1995; Wiegmann et al., 2011). Although the composition of Bibionomorpha remains controversial, in the strictest sense (following principally Wood & Borkent, 1989) it comprises 12 extant families of lower Diptera: Bibionidae, Bolitophilidae, Cecidomyiidae, Diadocidiidae, Ditomyiidae, Hesperinidae, Keroplatidae, Lygistorrhinidae, Mycetophilidae, Pachyneuridae, Rangomaramidae, and Sciaridae. Broader concepts of Bibionomorpha also include Anisopodidae *sensu lato* (including *Mycetobia* Meigen, 1818, *Olbiogaster* Osten-Sacken, 1886 and related genera in the sense of Michelsen, 1999), Canthyloscelidae, Scatopsidae, and sometimes even Axymyiidae and Perissommatidae (see e.g., Hennig, 1973; Oosterbroek & Courtney, 1995; Wiegmann et al., 2011; Lambkin et al., 2013). The principally fossil family Valeseguyidae (with one extant species), placed in Scatopsoidea by Amorim & Grimaldi (2006), belongs to Bibionomorpha as well. Additionally, several enigmatic genera that certainly belong to Bibionomorpha have not yet been definitely assigned to a family. These taxa were traditionally treated as the *Heterotricha* Loew, 1850 group but in recent years have been referred to as Sciaroidea *incertae sedis* (Chandler, 2002; Jaschhof, 2011; Hippa & Ševčík, 2014).

In terms of biodiversity, Bibionomorpha is a megadiverse group due to the inclusion of the fungus gnats (Sciaroidea, comprising the very large families Mycetophilidae and Sciaridae) and gall midges (family Cecidomyiidae), the latter presumably even being the most diverse and species-rich family of Diptera (cf. Hebert et al., 2016). The number of extant species of Bibionomorpha *sensu lato* currently described has been estimated at 15,000 (Pape, Bickel & Meier, 2009), although an inestimable number of species in this group still remain uncollected and undescribed. Moreover, fungus gnats and gall midges are notoriously abundant in trap catches (e.g., Malaise traps) from terrestrial habitats, especially mesic forests. Various subgroups of Bibionomorpha are the most speciose among fossil Diptera, being well represented in the fossil record since the Mesozoic and impressively documented from different ambers (Evenhuis, 1994; Blagoderov & Grimaldi, 2004; Grimaldi, Engel & Nascimbene, 2002; Hoffeins & Hoffeins, 2014).

The larval diets of Bibionomorpha are diverse, including detritophagy, saprophagy, predation, mycophagy and phytophagy. Mycophagy has been considered to be ancestral in Sciaroidea, and predation ancestral in Keroplatidae (Matile, 1997). However, these conclusions were based on relatively little empirical evidence and the biology of most Bibionomorpha, even on a generic level, remains understudied. As a notable exception, the biology of many phytophagous Cecidomyiidae has been studied in great detail (e.g., Gagné & Moser, 2013). As for adults, fungus gnats are certainly the most conspicuous bibionomorphs, since they are both abundant (usually aggregating in large numbers at the trunks of fallen, rotten trees, along stream banks, and at similar moist, shady places) and big enough to be noticed with the naked eye. Species of Bibionidae occurring in enormous numbers during spring are widely known, even among general naturalists, as March flies, or lovebugs.

In accordance with the significance of the group, the phylogenic relationships within Bibionomorpha have been studied many times, often with the aim to establish a natural family classification. Among the studies based on morphology are those by Hennig (1954), Hennig (1973), Rohdendorf (1964), Rohdendorf (1974), Rohdendorf (1977), Wood & Borkent (1989), Oosterbroek & Courtney (1995), Matile (1997), Fitzgerald (2004), Amorim & Rindal (2007) and Lambkin et al. (2013). Even so, the phylogenetic relationships within Bibionomorpha are still far from being clarified (e.g., Bertone, Courtney & Wiegmann, 2008). This is especially true in the Sciaroidea (including Bolitophilidae, Cecidomyiidae, Diadocidiidae, Ditomyiidae, Keroplatidae, Lygistorrhinidae, Mycetophilidae, Rangomaramidae, and Sciaridae) as several contradictory hypotheses have been proposed and debated in recent years (Matile, 1990; Matile, 1997; Chandler, 2002; Hippa & Vilkamaa, 2005; Hippa & Vilkamaa, 2006; Amorim & Rindal, 2007; reviewed by Jaschhof, 2011). As a result, one could get the impression that the morphology of adults (for various reasons larvae have not been studied in as much detail) cannot provide us with new and solid arguments in phylogenetic debates. For this reason, molecular approaches became the focus of Sciaroidea researchers, which seems natural considering that new characters are needed to advance the phylogenetic discussion and that the rapid development of molecular methods has raised great expectations. Molecular approaches have only recently been applied to phylogenetic studies of Bibionomorpha, but these studies focused either on certain subtaxa (e.g., Rindal, Søli & Bachmann, 2009; Ševčík, Kaspřák & Tóthová, 2013; Ševčík et al., 2014; Shin et al., 2013) or had a major focus beyond the infraorder (Bertone, Courtney & Wiegmann, 2008; Wiegmann et al., 2011; Beckenbach, 2012). As a consequence, taxon sampling was not adjusted to tackle the issues specific to Bibionomorpha.

There are a number of unresolved questions regarding Bibionomorpha phylogeny that we have aimed to address in this study:

Firstly, the delimitation of the infraorder (*sensu stricto versus sensu lato*) remains unclear, especially regarding the question whether families, such as Anisopodidae *sensu lato*, Scatopsidae, Canthyloscelidae, Axymyiidae, and Perissommatidae, belong to the Bibionomorpha or not.

Secondly, the phylogenetic position of non-sciaroid families, such as Bibionidae, Hesperinidae, and Pachyneuridae is still unresolved, in part due to the fact that hesperinid and pachyneurid specimens are rarely collected and poorly represented in collections. Representatives of these three families were included in the morphological analyses by both Fitzgerald (2004) and Amorim & Rindal (2007), which resulted in similar phylogenetic hypotheses, but these hypotheses still need to be tested, especially as the latter study was criticized for fundamental methodological shortcomings (Jaschhof, 2011).

Thirdly, the delimitation of the Sciaroidea is still a controversial issue, especially regarding the inclusion of the Cecidomyiidae and/or Ditomyiidae. Several studies, both molecular (Bertone, Courtney & Wiegmann, 2008) and morphological (Fitzgerald, 2004), have been unable to provide support for the monophyly of Sciaroidea (including Ditomyiidae) and most of the studies have not recognized Cecidomyiidae as a part of Sciaroidea.

Fourthly, and perhaps the most puzzling issue of all, is the phylogenetic position and assignment to family of the Sciaroidea *incertae sedis*, which is closely related to the question of how the family Rangomaramidae should be appropriately defined (cf. Jaschhof & Didham, 2002; Amorim & Rindal, 2007; Jaschhof, 2011). The authors of the present paper do not follow the concept of the Rangomaramidae as proposed by Amorim & Rindal (2007), a practice in common with the most recent Diptera Manuals (Brown et al., 2009; AH Kirk-Spriggs & BJ Sinclair, 2016, unpublished data) as well as papers specifically related to Sciaroidea *incertae sedis* (e.g., Hippa & Ševčík, 2014). Nevertheless, Amorim & Rindal’s (2007) results and proposals are discussed here when appropriate.

**Table 1:** List of specimens used for DNA extraction, with GenBank accession numbers. More information about the specimens is listed in the Table S1.

Taxa	12S	16S	18S	28S	COI	CAD
**MECOPTERA**						
*Microchorista philpotti*	KC177465	KC177459	KC177275	KC177635	HQ696580	n/a
*Nannochorista* sp.	n/a	n/a	n/a	n/a	n/a	KC177108
**DIPTERA**						
**Anisopodidae**						
*Mesochria cinctipes*	KP288699	KP288731	KP288776	KP288814	KT316866	n/a
*Mycetobia divergens*	KP288703	KP288735	KP288781	KP288818	KT316870	FJ040587
*Olbiogaster* sp.	KP288686	KX453698	n/a	KC177638	KT316855	KC177109
*Sylvicola fenestralis*	NC_016176	NC_016176	KC177287	KC177637	NC_016176	FJ040627
**Axymyidae**						
*Axymyia furcata*	n/a	n/a	n/a	KC177639	n/a	KC177110
*Protaxymyia thuja*	KP288702	KP288734	KP288780	KP288817	KT316869	KX453744
**Blephariceridae**						
*Edwardsina gigantea*	KC177470	KC177460	KC177283	KC177655	KC192960	FJ040624
*Liponeura cordata*	KX453689	KX453695	n/a	KX453707	KX453756	KX453718
**Bibionidae**						
*Bibio marci*	KJ136689	KJ136724	KP288758	KJ136761	KT316846	KX453730
*Dilophus femoratus*	KP288696	KP288728	KP288773	KP288811	KT316864	KX453740
*Penthetria funebris*	KP288689	KP288721	KP288767	KP288804	KT316858	KX453735
*Plecia nearctica*	KJ136690	KJ136725	KP288759	KJ136762	KT316847	n/a
**Bolitophilidae**						
*Bolitophila cinerea*	KJ136677	KJ136712	KP288741	KJ136749	KT316829	n/a
*Bolitophila occlusa*	KJ136678	KJ136713	KP288742	KJ136750	KT316830	n/a
*Bolitophilla tenella*	KP288698	KP288730	KP288775	KP288813	KT316836	n/a
**Canthyloscelidae**						
*Hyperoscelis veternos*a	KP288697	KP288729	KP288774	KP288812	KT316865	KX453741
*Synneuron annulipes*	KX453693	KX453701	n/a	KX453713	KX453763	n/a
*Synneuron decipiens*	n/a	n/a	n/a	KC177652	JN582256	n/a
**Cecidomyiidae**						
*Asphondylia sarothamni*	KP288692	KP288724	KP288770	KP288807	KX453761	KX453738
*Catocha angulata*	KP288677	KP288711	KP288750	KP288792	KT316837	KX453719
*Catotricha subobsoleta*	KP288706	KP288738	KP288784	KP288821	KT316873	KX453747
*Lasioptera rubi*	KP288691	KP288723	KP288769	KP288806	KT316860	KX453737
*Lestremia cinerea*	KJ136693	KJ136728	KP288761	KJ136765	KT316850	n/a
*Mayetiola destructor*	NC_013066	NC_013066	KC177284	KC177649	EU375697	FJ040625
*Porricondyla nigripennis*	KP288690	KP288722	KP288768	KP288805	KT316859	KX453736
**Chironomidae**						
*Chironomus tepperi*	NC_016167	NC_016167	KC177280	KC177658	NC_016167	FJ040616
**Culicidae**						
*Anopheles gambiae*	NC_002084	NC_002084	AM157179	KC177663	NC_002084	KC177121
**Deuterophlebiidae**						
*Deuterophlebia coloradensis*	n/a	n/a	GQ465776	FJ040539	GQ465781	KC177114
**Diadocidiidae**						
*Diadocidia valida*	KJ136699	KJ136735	KP288788	KJ136771	KT316877	KX453754
*Diadocidia ferruginosa*	KC435526	KC435562	KP288786	KC435598	KC435634	KX453752
*Diadocidia globosa*	KP288708	KP288740	KP288789	KP288822	KT316878	KX453755
**Ditomyiidae**						
*Asioditomyia* sp.	KP288678	KP288712	KP288751	KP288793	KT316838	n/a
*Ditomyia fasciata*	KJ136698	KJ136734	KP288787	KJ136770	KT316876	KX453753
*Symmerus annulatus*	n/a	KX453696	FJ171934	KX453708	KX453757	KC177112
**Dixidae**						
*Dixa submaculata*	KX453694	KX453702	KX453706	KX453714	KX453764	KC177123
**Hesperinidae**						
*Hesperinus brevifrons*	KP288705	KP288737	KP288783	KP288820	KT316872	KX453746
*Hesperinus ninae*	KP288687	KP288719	KP288765	KP288802	KT316856	KX453734
**Keroplatidae**						
*Arachnocampa flava*	NC_016204	NC_016204	KC177277	KC177644	NC_016204	KC178393
*Chiasmoneura anthracina*	KJ136682	KJ136717	KP288745	KJ136754	KT316833	n/a
*Keroplatus testaceus*	KJ136683	KJ136718	KP288746	KJ136755	KT316834	KX453716
*Macrocera centralis*	KP288682	KP288716	KP288755	KP288797	KT316841	KX453723
*Orfelia nemoralis*	KP288681	KP288715	KP288754	KP288796	KT316840	KX453722
*Robsonomyia* sp.	KP288683	n/a	KP288756	KP288798	KT316842	n/a
**Lygistorrhinidae**						
*Asiorrhina parasiatica*	KP288675	KP288709	KP288744	KP288790	KT316832	KX453715
*Blagorrhina* sp.	KP288694	KP288726	KP288772	KP288809	KT316862	KX453739
*Lygistorrhina*(*L*.) sp.	KP288693	KP288725	KP288771	KP288808	KT316861	KX453725
*Lygistorrhina (P.) cerqueirai*	KX453690	KX453697	KX453703	KX453709	KX453759	KX453724
**Mycetophilidae**						
*Exechia seriata*	KJ136688	KJ136723	KP288749	KJ136760	KT364885	n/a
*Gnoriste bilineata*	KP288679	KP288713	KP288752	KP288794	KT316839	KX453720
*Mycetophila alea*	KJ136687	KJ136722	KP288748	KJ136759	KJ136798	n/a
*Mycomya circumdata*	KJ136685	KJ136720	KP288747	KJ136757	KT316835	KX453717
*Rondaniella dimidiata*	KP288684	KP288717	KP288757	KP288799	KT316843	KX453726
*Sciophila geniculata*	KP288680	KP288714	KP288753	KP288795	KX453758	KX453721
**Pachyneuridae**						
*Cramptonomyia spenceri*	NC_016203	NC_016203	KP288779	KC177653	NC_016203	FJ040632
*Pachyneura fasciata*	KP288704	KP288736	KP288782	KP288819	KT316871	KX453745
**Psychodidae**						
*Clogmia albipunctata*	KP288695	KP288727	KC177281	KP288810	KT316863	FJ040622
*Lutzomyia longipalpis*	AY352674	n/a	AJ244428	FJ040565	GU909504	KC177125
*Phlebotomus papatasi*	EF613326	n/a	AJ391726	HM140979	JX105037	n/a
*Phlebotomus duboscqi*	EF613321	n/a	AJ627019	KC177666	KR020548	FJ040596
*Psychomora mycophila*	KP288701	KP288733	KP288778	KP288816	KT316868	KX453743
**Ptychopteridae**						
*Ptychoptera* sp.	NC_016201	NC_016201	GQ465777	FJ040542	NC_016201	KC177127
*Ptychoptera albimana*	KX453691	KX453699	KX453704	KX453711	KX453762	KX453750
**Scatopsidae**						
*Aspistes berolinensis*	KP288707	KP288739	KP288785	KX453710	n/a	KX453748
*Coboldia fuscipes*	KJ136692	KJ136727	KC177282	KJ136764	KT316849	FJ040623
*Scatopse notata*	KJ136691	KJ136726	KP288760	KJ136763	KT316848	KX453731
**Sciaridae**						
*Bradysia amoena*	GQ387651	GQ387651	KP288763	FJ040522	GQ387651	FJ040621
*Cratyna nobilis*	n/a	JQ613975	JQ613680	JQ613778	JQ613875	n/a
*Dolichosciara flavipes*	KJ136695	KJ136731	KP288762	KJ136768	KT316851	KX453732
*Sciara humeralis*	n/a	JQ613912	JQ613620	JQ613716	JQ613812	n/a
*Zygoneura sciarina*	KP288700	KP288732	KP288777	KP288815	KT316867	KX453742
**Simuliidae**						
*Parasimulium crosskeyi*	AF049472	n/a	n/a	KC177660	FJ524493	KC177118
**Tanyderidae**						
*Protoplasa fitchii*	KC177472	KC177462	KC177286	KC177670	NC_016202	FJ040626
**Thaumaleidae**						
*Thaumalea bezzii*	KX453692	KX453700	KX453705	KX453712	KT215925	KX453751
**Tipulidae**						
*Tipula abdominalis*	KC177466	KC177457	KC177288	KC177678	KC192958	GQ265584
**Trichoceridae**						
*Trichocera saltator*	GAXZ00000000.2	GAXZ00000000.2	GAXZ00000000.2	GAXZ00000000.2	GAXZ00000000.2	GAXZ00000000.2
***incertae sedis***						
*Chiletricha spinulosa*	KT316809	KT316814	KT316819	KT316824	KX453760	KX453727
*Insulatricha hippai*	KT316811	KT316816	KT316821	KT316826	KT316845	KX453729
*Nepaletricha sigma*	KJ136697	KJ136733	n/a	KP288800	KT316853	n/a
*Nepaletricha furcata*	KP288685	KP288718	KP288764	KP288801	KT316854	KX453733
*Ohakunea bicolor*	KT316810	KT316815	KT316820	KT316825	KT316844	KX453728
*Sciarosoma nigriclava*	KP288688	KP288720	KP288766	KP288803	KT316857	n/a
**Brachycera**						
*Asilus crabroniformis*	KC177475	KC177451	KC177289	KC177704	KC192962	EF650383
*Bombylius major*	KC177474	KC177450	KC177290	KC177708	KC192961	KC177144
*Ceratitis capitata*	NC_000857	NC_000857	KC177300	KC177754	NC_000857	XM_004529679.2
*Drosophila melanogaster*	X97155	KJ919952	KC177303	KC177803	HM102299	n/a
*Empis producta*	FJ808095	FJ808167	FJ808243	n/a	n/a	n/a
*Exeretonevra angustifrons*	KC177477	KC177453	KC177293	KC177685	KC192964	FJ040628
*Haematopota pluvialis*	KC177479	KC177454	KC177294	KC177694	KC192969	n/a
*Hermetia illucens*	KC177478	KC177455	KC177295	KC177682	GQ465783	n/a
*Megaselia scalaris*	KC177486	KC177442	KC177299	KC177721	KF974742	n/a
*Musca domestica*	AY573084	KC347601	KC177313	KC538816	JX438043	AY280689
*Physocephala marginata*	KC177481	KC177452	KC177304	KC177729	KM569879	HM062719
*Rachicerus* sp.	KT316813	KT316818	KT316823	KT316828	KT316875	KX453749
*Xylophagus ater*	KT316812	KT316817	KT316822	KT316827	KT316874	n/a

The present paper aims to provide answers, or partial answers, to the four questions outlined above using nuclear and mitochondrial gene markers and taxon sampling designed to address these Bibionomorpha-specific issues.

## Material and Methods

### Taxon sampling

A total of 94 terminal taxa are included in the dataset (Table 1 and Table S1). The ingroup contains 60 species from all the families traditionally placed in Bibionomorpha *sensu lato* (except Perissommatidae, Rangomaramidae *sensu* Jaschhof & Didham, 2002 and Valeseguyidae Amorim & Grimaldi, 2006) including six species of Sciaroidea *incertae sedis*. As outgroup taxa, we analysed 20 species from 13 families of non-bibionomorph lower Diptera, 13 species from 12 families of Brachycera, and one species of Mecoptera.

Samples containing these species were collected throughout the world, usually by means of Malaise traps, in the years 2006–2015 (Table S1). Most of the specimens were collected in localities where no field study permission is required. No taxon included in this study is specially protected by law. For the field research in Central Slovakia permission was issued by the Administration of Muránska planina National Park (No. 2011/00619-Ko and OU-BB-OSZP1-2014/13611-Ku). A part of the material examined was collected within the “Thailand Inventory Group for Entomological Research (TIGER) project” supported by the National Research Council of Thailand and the Department of National Parks, Wildlife and Plant Conservation, Thailand, who gave permission for research and the collection of specimens (see http://sharkeylab.org/tiger/). Two specimens used in the analyses were collected in Kuala Belalong Field Studies Centre, Brunei, in cooperation with the Institute for Biodiversity and Environmental Research, Universiti Brunei Darussalam, who also provided relevant permission (No. UBD-AVC-RI/1.24.1 and BioRIC/HOB/TAD/51-40).

All the specimens used for DNA extraction were identified by the authors (JŠ, SF, MJ), according to the most recent taxonomic literature. The voucher specimens are deposited in the reference collection of the Ševčík Lab, University of Ostrava, Czech Republic (JSL-UOC), with several specimens also deposited in the collection of the Silesian Museum, Opava, Czech Republic (SMOC); see Table S1.

Some sequences, mostly for outgroup taxa, were taken from the GenBank database. We were not able to obtain museum specimens of Rangomaramidae or Perissommatidae for our analyses so any efforts in the future to illuminate the phylogenetic position of these enigmatic groups by molecular approaches will depend on freshly collected material.

### DNA extraction, amplification and sequencing

The material used for DNA analysis was either alcohol-preserved (70% to 99.9% ethanol) or dried and pinned. The DNA was extracted using DNeasy Blood & Tissue Kit (Qiagen, Venlo, Netherlands) and NucleoSpin Tissue Kit (Macherey-Nagel, Düren, Germany) following both manufacturers’ protocols. Individual flies or tissue portions were rinsed in phosphate-buffered saline (PBS), placed in sterile Eppendorf tubes and incubated overnight at 56°C in lysis buffer with proteinase K. PCRs (total volume = 20 µl) were performed using primers for three mitochondrial genes: ribosomal 12S (Cook, Austin & Disney, 2004), ribosomal 16S (Roháček, Tóthová & Vaňhara, 2009), and protein-encoding COI (barcoding region, cf. Folmer et al., 1994); for three nuclear genes: ribosomal 28S (Belshaw et al., 2001) and 18S (Campbell, Ross & Woodward, 1995 or Katana et al., 2001), and two regions of the protein-encoding CAD (Moulton & Wiegmann, 2004), spanning positions 2200–3104 and/or 2977–3668 in the *Drosophila melanogaster* CAD sequence, see Table S2. We designed one additional primer to amplify the cytochrome oxidase I, mCOIa-R (5′-AAAATAGGGTCTCCTCCTCC-3′). In the case of the taxa *Pachyneura fasciata* (JSOUT37), *Asiorrhina parasiatica* (JSB4d), *Sciarosoma nigriclava* (JSOUT13b), and *Aspistes berolinensis* (JSOUT43), the 18S rRNA gene was amplified in two overlapping fragments. All amplified products were purified using QIAQuick PCR Purification Kit (Qiagen, Venio, Netherlands), GenElute™ PCR Clean-Up Kit (SIGMA) or Gel/PCR DNA Fragments Extraction Kit (Geneaid, New Taipei City, Taiwan). PCR products of CAD from several samples were extracted from agarose gel and purified with the Gel/PCR DNA Fragments Extraction Kit (Geneaid, New Taipei City, Taiwan).

Sequencing was carried out with BigDye Terminator ver.3.1 (Applied Biosystems, Foster City, USA) on an ABI 3100 genetic analysis sequencer (Perkin Elmer Applied Biosystems, Norwalk, CT, USA), or PCR products were sequenced by Macrogen Europe (Netherlands). All sequences were assembled, manually inspected, and primers trimmed in SEQUENCHER 5.0 (Gene Codes Corporation, Ann Arbor, USA). Sequences used to build an alignment for phylogenetic analysis were deposited in the GenBank database, accession numbers are listed in Table 1.

The identity of all the sequences was confirmed by BLAST similarity searches against NCBI database and double-checked in single gene trees to eliminate possible contaminations or other misleading results. Suspicious-looking sequences, where possible, were sequenced repeatedly from different specimens of the same species as a positive control (e.g., in *Asiorrhina parasiatica*, *Diadocidia globosa*, *Insulatricha hippai*, *Lygistorrhina* spp., *Ohakunea bicolor*, *Protaxymyia thuja*, *Sciarosoma nigriclava*).

### Sequence alignment and phylogenetic analyses

The ribosomal genes 12S, 16S, 18S and 28S were aligned using MAFFT version 7 (Katoh & Standley, 2013) on the MAFFT server (http://mafft.cbrc.jp/alignment/server/). Method L-INS-I, recommended for <200 sequences with one conserved domain and long gaps, was automatically selected by the program according to the alignment sizes. Resulting alignments were visually inspected and manually refined when necessary. The lengths of individual alignments were: 12S = 767 bp, 16 = 399 bp, 18S = 2,554 bp, 28S = 1,399 bp. We also tested some other alignment algorithms (Q-INS-I MAFFT—which considers secondary structure of the molecules; or T-Coffee), but none of them yielded a tree with better resolution.

All unreliably aligned regions were removed in the program GBLOCKS 0.91b (Castresana, 2000) on the Gblocks server (http://molevol.cmima.csic.es/castresana/Gblocks_server.html). Conditions have been set as follows: allowed smaller blocks, allowed gap positions within the final blocks and allowed less strict flanking positions. Sequences of the protein-coding genes COI and CAD were checked based on amino-acid translations and provided indel-free nucleotide alignments. The single-gene alignments were concatenated and the final concatenated alignment was deposited in the Figshare database (https://dx.doi.org/10.6084/m9.figshare.c.3458082.v1).

We made a comprehensive alignment of 94 terminal taxa comprising almost all major groups of the infraorder Bibionomorpha, as well as a number of outgroup taxa, including several representatives of Brachycera. We selected representatives of diverse lineages and various trophic strategies in each family of the ingroup to make up a balanced dataset. The final data matrix consisted of 5,018 characters: 12S—306 bp, 16S—326 bp, 18S—1,851 bp, 28S—452 bp, CAD—1425, COI—658 bp. Saturation analysis was performed for all six genes using Xia’s test implemented in DAMBE (Xia & Xie, 2001). In the case of the protein coding genes COI and CAD, saturation of third codon positions was tested separately.

Trees were rooted by the representative of the order Mecoptera, which is considered sister group to Diptera (e.g., Misof et al., 2014).

The dataset was analysed using Bayesian inference (BI) and maximum likelihood (ML) methods. The node support values are given in the form: posterior probability (PP) / bootstrap value (BV). We do not present here any results from the maximum parsimony analysis, because it provided almost no significant support at the higher taxonomic levels (interfamilial relationships), in concordance with theoretical arguments indicating the limited performance of the MP method in molecular phylogenetics (e.g., Gadagkar & Kumar, 2005; Spencer, Susko & Roger, 2005).

To evaluate the best fit model for the BI and ML analyses, the concatenated dataset was partitioned into six sets: six gene regions (12S = 1–306, 16S = 307–632, 18S = 633–2,483, 28S = 2,484–2,935, CAD = 2,936–4,360, COI = 4,361–5,018). Each of the partitions was evaluated in MrModeltest v.2.2 (Nylander, 2004) using both hierarchical likelihood ratio tests (hLRTs) and Akaike Information Criterion (AIC). We used model GTR + Γ + I (Rodriguez et al., 1990) for Bayesian inference and GTR + Γ for ML analysis.

The partitioned Bayesian inference of 50 million generations on the concatenated dataset was implemented in MrBayes version 3.2.2 (Huelsenbeck & Ronquist, 2001) and carried out on the CIPRES computer cluster (Cyberinfrastructure for Phylogenetic Research; San Diego Supercomputing Center, Miller, Pfeiffer & Schwartz, 2010). Burn-in was set as 30%.

The ML analyses were conducted on the CIPRES computer cluster using RAxML-HPC BlackBox 7.6.3 (Stamatakis, 2006) employing automatic bootstrapping on single gene alignments to check potential conflicting topologies and other artifacts. Subsequently, the same algorithm was applied on the concatenated partitioned dataset.

Phylogenetic trees were visualized using Interactive Tree Of Life (iTOL) (Letunic & Bork, 2011). The trees presented are Bayesian or ML topologies with node support values from both the analyses.

## Results and Discussion

### Comparison of the Bayesian and maximum likelihood analyses

**Figure 1:**
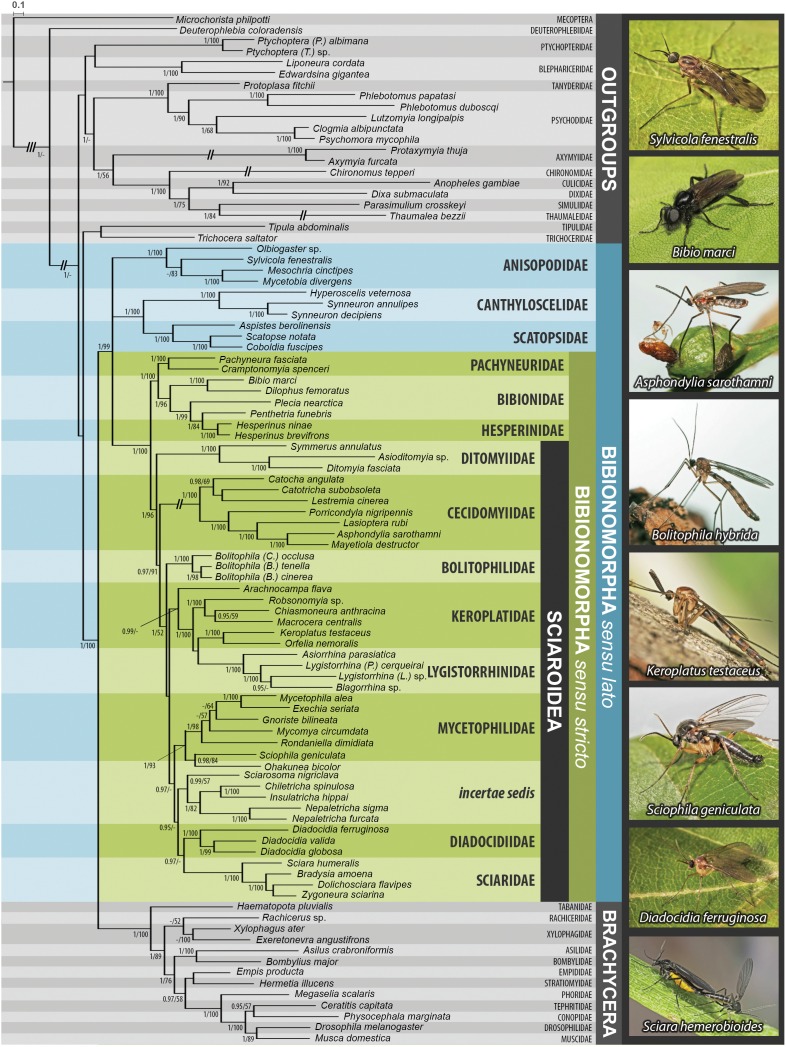
Bayesian hypothesis for relationships among selected taxa of Bibionomorpha based on DNA sequence data (18S, 28S, CAD, 12S, 16S, and COI), 5,018 characters. Support numbers refer to posterior probability over 0.95/ bootstrap value over 50. The branches marked as “//” have been shortened to its half to fit them into the graphic. All photographs by J Ševčík.

**Figure 2:**
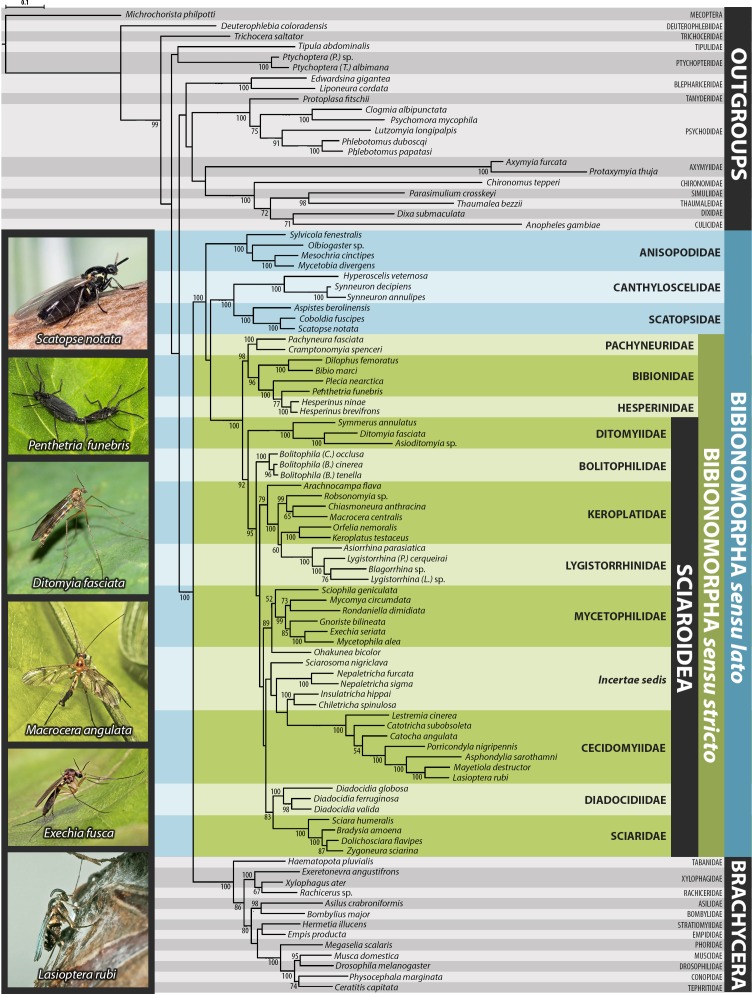
Maximum likelihood hypothesis for relationships among selected taxa of Bibionomorpha based on dataset without 3rd codon positions. Support numbers refer to bootstrap values over 50. Photographs by J Roháček (*Scatopse notata*) and J Ševčík.

**Figure 3:**
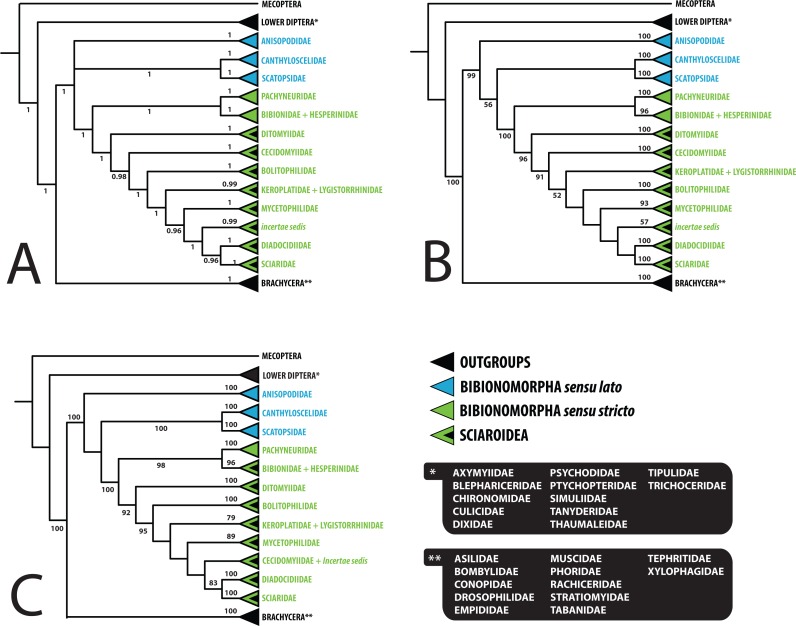
Simplified comparison of relationships among the families of Bibionomorpha. (A) Bayesian tree; (B) Maximum likelihood tree; (C) Maximum likelihood tree based on dataset without saturated 3rd codon positions. Support numbers refer to posterior probability over 0.95 and bootstrap value over 50, respectively.

The results based on Bayesian inference (BI) and maximum likelihood (ML) analyses of the dataset are summarized in Figs. 1–3, Figs. S1 and S2. For BI, standard deviation of split frequencies was in all cases <0.01. The log likelihood value for the best tree of the dataset was −128022.18. Both the model-based methods (BI, ML) yielded mostly congruent nodes, with several exceptions mostly at the family level (Figs. 1–3). Well-supported relationships were consistent across all the trees obtained.

Saturation analyses revealed that the phylogenetic markers used in this study show a rather low level of saturation, with the exception of the third positions of the protein coding genes COI and CAD. However, ML phylogenetic analysis based on the dataset with excluded third codon positions provided a tree with very similar topology (cf. Figs. 1 and 2) in comparison with the original tree, with small differences only within several family-level clades and also slight changes in node support values. These minor differences are discussed below when relevant.

### Delimitation, monophyly and affiliation of Bibionomorpha

The monophyly of Bibionomorpha *sensu stricto* (containing Sciaroidea, Bibionidae, Hesperinidae, and Pachyneuridae) was established with high support (PP = 1.00/BV = 100) in both the model-based analyses (see Figs. 1–3, Figs. S1 and S2). The monophyly of Bibionomorpha *sensu lato* (containing Bibionomorpha *s. str.* plus Anisopodidae *sensu lato*, Canthyloscelidae, and Scatopsidae, 1.00/99) was also established. These findings are consistent with results from the previous molecular and morphological studies, which usually found Bibionomorpha (in various concepts) to be monophyletic (e.g., Hennig, 1954; Oosterbroek & Courtney, 1995; Amorim & Yeates, 2006; Bertone, Courtney & Wiegmann, 2008; Wiegmann et al., 2011). Amorim & Yeates (2006) even suggested raising the Bibionomorpha to the rank of suborder.

The present study shows Bibionomorpha *sensu lato* to be sister group to Brachycera (1.00/99), which is congruent with results from the molecular studies by both Bertone, Courtney & Wiegmann (2008) and Wiegmann et al. (2011). Hennig (1973), who came to the same result through morphological study, referred to two synapomorphies supporting this relationship: the conspicuous enlargement of the second latero-tergite and the undivided postphragma of the thorax. Fitzgerald (2004) considered the structure of the parameres (presence of a dorsal sclerite and ventrolateral apodemes) as synapormorphies of this taxon and Michelsen (1996) even introduced a new taxon, Neodiptera, for Bibionomorpha *sensu lato* + Brachycera based on several cervical and prothoracic skeleto-muscular characters as synapomorphies.

On the other hand, the Bayesian tree by Beckenbach (2012), based on the complete mitochondrial genome of 24 species of Diptera, revealed Anisopodidae + Tanyderidae to be the sister group to Brachycera, but with relatively low support. The morphological study by Oosterbroek & Courtney (1995) considered only Anisopodidae (rather isolated in their tree from the rest of Bibionomorpha) as the sister group to Brachycera, with Anisopodidae + Brachycera being the sister group to the clade containing Tipulidae and Trichoceridae. Their Bibionomorpha included the family Axymyiidae and formed a sister clade to the whole “higher Nematocera and Brachycera” (Perissommatidae, Scatopsidae, Synneuridae, Psychodidae, Tipulidae, Trichoceridae, Anisopodidae, and Brachycera).

Within the Bibionomorpha *sensu lato*, all the major lineages (i.e., Anisopodoidea, Scatopsoidea, Bibionoidea, and Sciaroidea) were highly supported as monophyletic groups in both model-based analyses (1.00/100, 1.00/100, 1.00/100, and 1.00/96, respectively, see Figs. 1–3).

### Delimitation and monophyly of Sciaroidea

The monophyly of fungus gnats, Sciaroidea (= Mycetophiliformia of Amorim & Rindal, 2007), was confirmed here with high support (1.00/96), with both Cecidomyiidae and Ditomyiidae being integral parts of Sciaroidea. Previous phylogenetic studies based on morphology mostly considered Cecidomyiidae as the sister group to Sciaroidea (cf. Matile, 1990; Matile, 1997; Søli, 1997; Chandler, 2002; Hippa & Vilkamaa, 2005; Hippa & Vilkamaa, 2006). Amorim & Rindal (2007) used the name Mycetophiliformia for the large assemblage of taxa grouped into 5 superfamilies: Cecidomyioidea (containing Cecidomyiidae), Sciaroidea (containing only Sciaridae), Rangomaramoidea (Rangomaramidae in a broad sense, including most of the *incertae sedis* genera), Keroplatoidea (Ditomyiidae, Bolitophilidae, Diadocidiidae, Keroplatidae) and Mycetophiloidea (Lygistorrhinidae, Mycetophilidae). The trees they presented differ from our results in many ways but in all of them Cecidomyiidae form a sister branch to the rest of their Mycetophiliformia. Oosterbroek & Courtney (1995) considered Cecidomyiidae + Sciaridae as the sister group to Mycetophilidae *sensu lato*.

It is beyond the scope of this study to discuss the details of all these morphological analyses, but we think that a general failure of the past is underappreciation of the relevance of Catotrichinae, which according to the prevailing opinion (cf. Gagné & Jaschhof, 2014) are sister group to all the other Cecidomyiidae. The subfamily Catotrichinae contains only a few species, which are seldom treated in publications (most recently by Jaschhof & Jaschhof (2008), so too late to be taken in consideration by the studies referred to above). Even more important, hardly any dipterist has seen specimens of Catotrichinae so as to appreciate their remarkable resemblance with fungus gnats rather than with other gall midges. As another problem, knowledge of Cecidomyiidae morphology is evidently often too vague to interpret characters and character states correctly (cf. Hippa & Vilkamaa, 2005; Amorim & Rindal, 2007).

The molecular analysis by Bertone, Courtney & Wiegmann (2008) recovered a paraphyletic Sciaroidea, with Ditomyiidae excluded, and with moderate support 0.92/76 for a clade consisting of Ditomyiidae + (Bibionidae + Pachyneuridae). The incongruence between the results of Bertone, Courtney & Wiegmann (2008) and our results might possibly be explained by the more extensive taxon sampling (almost three times as many species of Bibionomorpha *sensu lato* included) in the present study.

### Monophyly and interrelationships of bibionomorph families

Although the sampling was not designed to thoroughly answer questions on the monophyly of particular families, most of the previously recognized families of Bibionomorpha were monophyletic with high support in all the analyses performed (Figs. 1–3, Figs. S1 and S2). The paraphyly of Keroplatidae is discussed below. The paraphyly of Mycetophilidae (with respect to *Ohakunea* Edwards, a genus usually regarded as unplaced to family) as suggested in Fig. 1 is surprising and is also commented on below.

The position of Anisopodidae *sensu lato* and Scatopsoidea (Scatopsidae + Canthyloscelidae) within Bibionomorpha *sensu lato* was not resolved in the Bayesian tree (Fig. 1). However, in the ML trees (Fig. 2, Figs. S1 and S2), Scatopsoidea was found to be the sister group to Bibionomorpha, with Anisopodidae being sister group to Bibionomorpha *sensu stricto* + Scatopsoidea, in concordance with previous major studies (Bertone, Courtney & Wiegmann, 2008; Wiegmann et al., 2011; Lambkin et al., 2013).

With respect to the position of Axymyiidae, our analysis produced a surprising result. While some previous studies placed Axymyiidae as the sister group to Bibionomorpha *sensu stricto* (e.g., Oosterbroek & Courtney, 1995; Wiegmann et al., 2011) or in a clade together with Pachyneuridae and Perissommatidae (e.g., Hennig, 1973; Amorim, 1993), our study grouped Axymyiidae with Culicomorpha, though with only moderate support (1.00/56). To exclude the possible long-branch attraction artifact in this case, we tested the ML topology after removal of the long-branch forming Culicomorpha clade. The resulting topology confirmed the position of Axymyiidae within the outgroup taxa. A similar topology was introduced by Bertone, Courtney & Wiegmann (2008), with Culicomorpha being the sister group to Axymyiidae + Nymphomyiidae. The morphological study by Fitzgerald (2004) also found Axymyiidae neither within nor sister group to Bibionomorpha. While several studies seem to indicate that Axymyiidae may not belong within, or as sister group to, Bibionomorpha, the phylogenetic position of Axymyiidae clearly requires further study.

As regards the families of Sciaroidea, BV supports from our ML analysis are generally rather low to infer interfamilial relationships, but PP supports for our Bayesian analysis provide better resolution. Concerning the relationships between families within Sciaroidea, the only well supported node in both the model-based analyses (1.00/100) is Keroplatidae, excluding *Arachnocampa* Edwards, 1924, and containing Keroplatinae, Macrocerinae and Lygistorrhinidae; see Figs. 1 and 2 and ‘Paraphyly of Keroplatidae’.

Ditomyiidae was shown to branch basally from the rest of Sciaroidea, with high support in both the analyses (1.00/96). This result is supported by most previous studies based on morphology (Zaitzev, 1983; Zaitzev, 1984; Wood & Borkent, 1989; Matile, 1990; Matile, 1997; Hippa & Vilkamaa, 2006), pointing to several primitive characters of ditomyiid larvae, such as the presence of the eighth abdominal spiracle and the structure of the mouthparts.

The family Cecidomyiidae is supported as the sister group to the remainder of Sciaroidea (excluding Ditomyiidae) in both the Bayesian and ML analyses (0.97/91). We consider it remarkable that our analyses showed Cecidomyiidae to be firmly nested within the fungus gnats, as discussed above. Interestingly, in the tree based on 18S only, as well as in the tree based on ribosomal genes only, Cecidomyiidae appeared in the clade with two *incertae sedis* genera (*Chiletricha* Chandler + *Insulatricha* Jaschhof) with high support in the ML analysis (BV = 100), indicating possible affinities of these groups. Also, in the ML tree based on the dataset with the saturated third codon positions removed (Fig. 2), Cecidomyiidae and the *incertae sedis* merged into one clade (albeit poorly supported). It will be very interesting to see how the topology obtained here will change when other unplaced Sciaroidea, as well as *Rangomarama* (discussed by Jaschhof & Didham (2002) to be the sister group of Cecidomyiidae), can be added to the dataset. The topologies proposed by Matile (1990) and Matile (1997) differ from our results mainly in the positions of Ditomyiidae, Diadocidiidae and Cecidomyiidae. Some previous authors (e.g., Oosterbroek & Courtney, 1995; Wood & Borkent, 1989) considered Cecidomyiidae as a sister group to Sciaridae. This view is not supported by our analyses.

Bolitophilidae is supported as the sister group to the other Sciaroidea (excluding Ditomyiidae and Cecidomyiidae), but with high support only in our Bayesian analysis (1.00/52). This clade was also supported as monophyletic by a number of larval synapomorphies by Fitzgerald (2004: 42) though larval stages of many taxa remain unknown and were therefore not included. Bolitophilidae is a family poorly represented in previous phylogenetic studies, especially molecular ones. As an exception, Wiegmann et al. (2011) included one species of *Bolitophila* Meigen in their dataset. Their analysis resulted in the sister grouping of Bolitophilidae and Keroplatidae, a topology that differs from ours, indicating the need of further studies into this issue in the future. One of the possible reasons for this discrepancy might be the fact that *Bolitophila* sp. was represented in their dataset by only three gene markers (28S,TPI, AATS1) and the whole family Keroplatidae only by one rather atypical member, *Arachnocampa flava* Harrison, 1966. The possible relationship of *Arachnocampa* to Bolitophilidae is an interesting hypothesis itself, though beyond the scope of this paper, considering the fact that the type species of the genus, *Arachnocampa luminosa* (Skuse, 1891) was initially described in *Bolitophila* and removed from this genus subsequently by Edwards (1924), who also discussed similarities and differences of the two genera.

The present study corresponds with an earlier one by Ševčík et al. (2014) which suggested Sciaridae to be the closest relative of Diadocidiidae, although with relatively high support only in Bayesian analysis (0.96/-). However, this clade is better supported (1/74) in the tree without the *incertae sedis* genera (Fig. S1) and in the ML tree based on the dataset without saturated third codon positions (Fig. 2).

Likewise, the topology (Diadocidiidae + Sciaridae) + Sciaroidea *incertae sedis* is moderately supported but only in our Bayesian analysis (PP = 0.95). There is slightly better support (1/52) for the sister grouping found for the entire latter clade + Mycetophilidae including *Ohakunea* (Fig. 1).

As revealed in this study, the relationships among the families of Sciaroidea still require further testing with the addition of more *incertae sedis* taxa and with a more extensive gene sampling (e.g., from next-generation sequencing). However, judging from the short branches in the family-level clades, it can be concluded that these taxa underwent very rapid radiation leaving low phylogenetic signal, which will be difficult to resolve. Another serious limitation will always be obtaining fresh specimens with a sufficient amount of DNA/RNA for several key taxa that are extremely rare.

### Paraphyly of Keroplatidae

The family Keroplatidae is paraphyletic with respect to Lygistorrhinidae in this study in both BI and ML analyses (the node support for the entire clade is 0.99/47, see Fig. 1) and also in the ML analysis based on the dataset without the saturated third codon positions (Fig. 2). The close relationship of Keroplatidae and Lygistorrhinidae was also indicated in most of the single-gene trees, being best supported in the CAD-based tree.

The sister clade to *Arachnocampa*, containing all the remaining taxa of Keroplatidae and Lygistorrhinidae, is even better supported (1.00/100). Within the latter clade, the genera of Lygistorrhinidae form a sister group to Keroplatinae, but with low support (0.65/46), see also below (‘Keroplatidae and Lygistorrhinidae’).

Tuomikoski (1966) was the first to hypothesize a possible relationship between Lygistorrhinidae and Keroplatidae, indicating that *Lygistorrhina* Skuse, 1890 most probably represents only a specialized group within Keroplatidae. He argued that the reduced wing venation in this genus may be misleading and that the thoracic structure, male terminalia, and other features of *Lygistorrhina* are very similar to those of *Fenderomyia* Shaw, 1948 and other taxa of Macrocerinae. However, his views were criticized by Thompson (1975), arguing that some of the characters shared by both groups (e.g., simple type of the male terminalia) are actually symplesiomorphies. Subsequent studies based on morphology have usually placed Lygistorrhinidae close to Mycetophilidae, and Keroplatidae close to Diadocidiidae (Matile, 1990; Matile, 1997; Chandler, 2002; Hippa & Vilkamaa, 2006; Amorim & Rindal, 2007). On the contrary, the close relationship between Lygistorrhinidae and Keroplatidae was indicated by three independent previous molecular studies (Bertone, Courtney & Wiegmann, 2008; Rindal, Søli & Bachmann, 2009; Ševčík et al., 2014) and it has also recently been suggested by Kallweit (2014) based on morphology.

The results of our analyses thus strongly support the inclusion of Lygistorrhinidae in the family Keroplatidae, probably best treated as a subfamily of the latter. The position of *Arachnocampa*, as discussed above (in ‘Monophyly and interrelationships of bibionomorph families’), requires further study, which is beyond the scope of this paper.

### Position of Sciaroidea incertae sedis

The genera *Chiletricha, Insulatricha, Nepaletricha* Chandler, 2002, *Ohakunea* and *Sciarosoma* Chandler, 2002, currently not assigned to a family, have usually been treated within the *Heterotricha* group (Chandler, 2002) or Sciaroidea *incertae sedis* (Jaschhof, 2011). Notable exceptions are Hippa & Vilkamaa (2005), who hypothesized *Sciarosoma* (in a subfamily of its own, Sciarosominae) as belonging to the Sciaridae, and Amorim & Rindal (2007), who placed all these genera except *Sciarosoma* in the broadly defined family Rangomaramidae (*Sciarosoma* was left unplaced to family within their Mycetophiliformia = Sciaroidea). Both these studies were challenged in the past (Jaschhof et al., 2006; Jaschhof, 2011, respectively). Most recently, Hippa & Ševčík (2014) discussed possible inclusion of *Nepaletricha* and related genera in the fossil family Antefungivoridae, based on the similarity of the wing venation.

These five genera are the only taxa from this enigmatic grouping with molecular data currently available. Our analyses showed *Ohakunea* to be only distantly related to the other four genera (Fig. 1), even though its position within the Mycetophilidae came as a surprise (see also ‘Mycetophilidae’). The other enigmatic genera are united in a single moderately supported clade (0.99/57) and as sister group to the clade Diadocidiidae + Sciaridae, but with lower support (0.95/-). Within this clade, *Sciarosoma* is branching basally to the well supported (1.00/82) clade of *Nepaletricha* and *Chiletricha* + *Insulatricha*.

As long as the majority of *incertae sedis* remain unstudied, we think it premature to attach too much importance to the specific positions of the enigmatic genera in the topology presented here, but the well-supported monophyly of (*Chiletricha* + *Insulatricha*) + *Nepaletricha* could be interpreted as giving support to the Chiletrichinae of Amorim & Rindal (2007).

When we excluded all the *incertae sedis* genera from the dataset (Fig. S1), the support values for the clade Diadocidiidae + Sciaridae increased to 1.00/78 and those for the Keroplatidae clade (including *Arachnocampa* and Lygistorrhinidae) increased to 1.00/60. This manipulation shows how important it is to include as many of the *incertae sedis* genera as possible in future molecular analyses.

### Relationships within particular families

#### Anisopodidae

The monophyly of Anisopodidae *sensu lato* is well supported (1.00/100) in all the analyses (Figs. 1 and 2), as well as the sister relationship of *Mycetobia* Meigen, 1818 and *Mesochria* Enderlein, 1910. The topology *Olbiogaster* + (*Sylvicola* + *Mycetobia*) in our analyses is in concordance with the hypothesis based on morphology given by Amorim & Tozoni (1994) while Michelsen (1999) suggested a different relationship, *Sylvicola* Harris, 1780 + (*Olbiogaster* Osten-Sacken, 1886 + Mycetobiidae). The latter topology was produced by our datasets with the third codon positions removed (Fig. 2) but with very low support for the clade (*Olbiogaster* + Mycetobiinae). A comprehensive molecular phylogeny of this group, based on better taxon sampling, is needed to elucidate these relationships.

#### Bibionidae and Pachyneuridae

The monophyly of Bibionidae *sensu lato* (Bibionidae *sensu stricto* + Hesperinidae) was established with high support (1.00/96). The clade containing the genera *Bibio* Geoffroy, 1762 and *Dilophus* Meigen, 1803 (Bibioninae) is sister group to the clade *Plecia* Wiedemann, 1828 + (*Penthetria* Meigen, 1803 + *Hesperinus* Walker, 1848), with all nodes well supported (Fig. 1). This topology suggests that either *Hesperinus* should not be classified in the separate family Hesperinidae because it would render the rest of Bibionidae paraphyletic or, Bibioninae, *Plecia*, *Penthetria*, and *Hesperinus* each need to be treated as distinct families. The position of *Hesperinus* within (rather than sister group to) Bibionidae is a novel hypothesis (DNA sequences for *Hesperinus* are published here for the first time) and it conflicts with several previous hypotheses that place *Hesperinus* as sister group to the remaining Bibionidae (Blaschke-Berthold, 1994; Fitzgerald, 2004; Pinto & Amorim, 2000). The topology found here requires a number of morphological characters that have been found to support bibionids exclusive of *Hesperinus* (e.g., male eye holoptic, male antennal flagellomeres compressed, larva with fleshy tubercles, larval intersegmental fissures separating abdominal segments seven and eight unaligned laterally, and larval abdominal segment three with three pseudosegments, see Fitzgerald, 2004) to be independently derived multiple times or derived once and then secondarily lost in *Hesperinus*. Further studies to elucidate these issues are thus warranted. Congruent with previous morphological studies focused on bibionids (Fitzgerald, 2004; Pinto & Amorim, 2000) the clade *Plecia* + *Penthetria* was not supported in this analysis thereby further discouraging the recognition of this clade which is commonly classified as Pleciidae or Pleciinae.

Bibionoidea (Bibionidae *sensu lato* + Pachyneuridae) was strongly supported as a monophyletic group in our analyses (1.00/100) echoing the findings of several earlier morphological (Blaschke-Berthold, 1994; Fitzgerald, 2004) and molecular studies (Bertone, Courtney & Wiegmann, 2008; Wiegmann et al., 2011). Due to the rarity of pachyneurids for study, the monophyly of the family Pachyneuridae *sensu lato* (*Pachyneura* Zetterstedt, 1838 + Cramptonomyiinae) has only rarely been assessed (Amorim, 1993; Blaschke-Berthold, 1994; Fitzgerald, 2004) and monophyly of the group has never been tested using molecular characters (*Pachyneura* is here sequenced for the first time). Fitzgerald (2004: 41) found seven unambiguous characters supporting monophyly of Pachyneuridae *s*.*l*., including one adult and three larval characters unique to the family. The present study also found strong support for the monophyly of Pachyneuridae *s*.*l*. thereby not supporting the previous hypothesis based on wing venation (Amorim, 1993) that considers Pachyneuridae *s*.*l*. to be paraphyletic and treats the genus *Pachyneura* in Axymyiomorpha with Axymyiidae.

#### Cecidomyiidae

The monophyly of Cecidomyiidae is here fully supported (1.00/100) in all the analyses (Figs. 1–3, Figs. S1 and S2). All the major lineages (subfamilies) recognised within the family except Winnertziinae are represented in our dataset. The clade *Catocha* Haliday, 1833 + (*Catotricha* Edwards, 1938 + *Lestremia* Macquart, 1826) was found to be the sister branch to the other Cecidomyiidae, which is remarkable insofar as solely Catotrichinae are usually placed at the base of the family (see above). The sister branch is formed by *Porricondyla* Rondani, 1840 + (*Asphondylia* Loew, 1850 + (*Lasioptera* Meigen, 1818 + *Mayetiola* Kieffer, 1896)), which is congruent with data based on morphology (Gagné, 1989; Jaschhof, 2001; Jaschhof & Jaschhof, 2009; Jaschhof & Jaschhof, 2013).

A molecular phylogeny of Cecidomyiidae, with taxon sampling that better represents the full range of this diverse family, is in preparation (T Sikora et al., 2016, unpublished data).

#### Keroplatidae and Lygistorrhinidae

The paraphyly of Keroplatidae with respect to Lygistorrhinidae was discussed above. Within the family Lygistorrhinidae, *Asiorrhina* Blagoderov, Hippa & Ševčík (2009) is sister group to the other Lygistorrhinidae (Fig. 1 and Fig. S1, 1.00/100), similar to the phylogenies proposed by Blagoderov, Hippa & Ševčík (2009), Hippa, Mattsson & Vilkamaa (2005) and Ševčík et al. (2014).

Within the Keroplatidae, *Arachnocampa* is sister group to the rest of Keroplatidae and Lygistorrhinidae (but see the discussion above, in ‘Monophyly and interrelationships of bibionomorph families’). Two major subclades, corresponding to the subfamilies of Matile (1990), can also be distinguished: Keroplatinae and Macrocerinae. Both of these groups were well supported (Fig. 1, 1.00/100, resp. 1.00/100) but the sister group relationship between Keroplatinae and Lygistorrhinidae is only poorly supported (0.65/46). The exact position of the subfamily Sciarokeroplatinae (see Papp & Ševčík, 2005) could not be ascertained by molecular methods because no fresh specimens of its only genus, *Sciarokeroplatus* Papp & Ševčík, 2005, have been available to study.

#### Mycetophilidae

The monophyly of Mycetophilidae is well supported (1.00/94), but only in the tree without the *incertae sedis* genera (Fig. S1). In the tree in Fig. 1, surprisingly, *Ohakunea* nested within the mycetophilid clade (1.00/93) as sister branch to *Sciophila* Meigen, 1818 (0.98/84). Its position clearly demonstrates the paraphyly of the *incertae sedis* genera but the possible relationship with *Sciophila* (or other mycetophilid genera) is difficult to substantiate by morphological characters (except the macrotrichia on the wing membrane and other plesiomorphic features). Considering that the known distribution of *Ohakunea* is restricted to the Australasian and Neotropical regions, it was concluded that it may represent an ancient lineage of sciaroids that evolved in Gondwanaland (Jaschhof & Hippa, 2003). The surprising position of *Ohakunea* within the Mycetophilidae clade may also be attributed to the relatively low taxon sampling of Mycetophilidae in this study; with the addition of more genera, its position could change. It is worth mentioning in this context that in the tree based on the dataset without the saturated third codon positions (Fig. 2), *Ohakunea* appeared as sister group to the family Mycetophilidae. Clearly, this issue requires a separate study.

Within the family Mycetophilidae, Sciophilinae (represented here by its type genus *Sciophila*) is usually considered to show plesiomorphic character states while subfamily Mycetophilinae, here represented by the genera *Exechia* Winnertz, 1863 and *Mycetophila* Meigen, 1803, is usually considered a more advanced group (e.g., Søli, 1997), sharing several apomorphic character states (e.g., wing microtrichia arranged in regular rows, structure of proepimeron and epimeron, and strict endomycophagy of the larvae). This view is also supported by our data (Figs. 1, 2 and Figs. S1, S2).

#### Sciaridae

A comprehensive molecular phylogeny of this family was recently presented by Shin et al. (2013). The sampling of Sciaridae in our study is necessarily limited to keep the numbers of terminal taxa in proportion for particular families, but the relationships among the genera included in our dataset principally agree with the topology presented in the latter study (Figs. 1, 2 and Figs. S1, S2).

### Comments on the selection of molecular markers and perspectives for the future

This study represents the first molecular phylogeny of Bibionomorpha based on a comprehensive taxon sampling. We tried to use easily-amplifiable, traditional gene markers because we were strongly limited by the scarcity of many taxa included in the dataset (and therefore had a shortage of DNA). For the following rare genus-group taxa we publish the first molecular data at all: all the Sciaroidea *incertae sedis* included (except *Nepaletricha*), *Asioditomyia* Saigusa, 1973, *Blagorrhina* Hippa, Mattison & Vilkamaa, 2009, *Catocha*, *Catotricha*, *Chiasmoneura* Meijere, 1913, *Hesperinus*, *Hyperoscellis* Hardy & Nagatomi, 1960, *Mesochria*, *Pachyneura*, *Protaxymyia* Mamaev & Krivosheina, 1966, *Robsonomyia* Matile & Vockeroth, 1980, *Rutylapa* Edwards, 1929—and for some clades we thus present the first phylogenetic hypotheses based on molecular data.

We also succeeded in incorporating two regions (of total length more than 1,400 bp) of the protein-coding nuclear gene CAD, which is recommended for use in reconstruction of Diptera phylogeny by Moulton & Wiegmann (2004) and subsequent authors. The amplification of the various fragments of this gene proved to be much more difficult than in traditional markers, so the inclusion of more protein-coding nuclear genes has not been possible with current material, which includes many rare species, available as only one or two specimens, usually collected several years ago. As soon as more fresh material (especially of Sciaroidea *incertae sedis*) is available for next-gen sequencing, which may take several years or more, it should be possible to design specific primers for Sciaroidea and amplify the supposedly more informative protein-coding nuclear genes, such as MCS and MAC (see Winkler et al., 2015). Our attempts to amplify MCS and MAC markers from most of the specimens of Bibionomorpha using the primers designed for other groups of Diptera failed.

Despite these shortcomings, we consider it important to publish a tree with lower resolution in a few branches because it contributes to our understanding of the performance of these traditional markers across the various taxa of Bibionomorpha, especially when they provide sufficient resolution for most other relationships within the group. Our dataset is largely based (74% of base pairs) on the very little saturated nuclear 18S, 28S and CAD genes, more suitable for deeper relationships, in combination with three short mitochondrial regions included to improve the resolution of terminal branches, and in the case of COI also to enrich the DNA barcode database.

## Conclusions

1. Monophyly of Bibionomorpha is supported in both the strict sense (Sciaroidea + Bibionoidea) and the broad sense (Bibionomorpha *sensu strico* + Anisopodoidea + Scatopsoidea). Axymyiidae is not resolved as part of, or as the sister group to, Bibionomorpha.
2. The monophyly of the groups Bibionoidea (Bibionidae *sensu lato* + Pachyneuridae *sensu lato*), Bibionidae *sensu lato* (Bibionidae *sensu stricto* + Hesperinidae), and Pachyneuridae (*Pachyneura* + Cramptonomyiinae) were all established with high support in all the analyses. Based on the topology recovered here, treating *Hesperinus* as the distinct family Hesperinidae would render the rest of Bibionidae paraphyletic. Diverging from previous hypotheses that place *Hesperinus* as the sister group to the remaining bibionids, the position of *Hesperinus* in this study is a novel hypothesis that requires further testing. As in previous studies, Pleciidae or Pleciinae (*Plecia* + *Penthetria*) was not supported as a monophyletic group and its usage should be discontinued.
3. The major lineages of Bibionomorpha *sensu lato* (Sciaroidea, Bibionoidea, Anisopodoidea, and Scatopsoidea) are well supported as monophyletic groups and all included families are supported as monophyletic with the exception of Keroplatidae and Bibionidae *sensu lato*. Both Cecidomyiidae and Ditomyiidae were recovered in model-based analyses as a part of Sciaroidea with high support. All the other families previously placed in Sciaroidea were confirmed to belong to this monophyletic group. The Keroplatidae clade (including Lygistorrhinidae) is well supported, but not so much with *Arachnocampa*. In most other cases support is too low to confidently resolve family level relationships within Sciaroidea.
4. The five genera of Sciaroidea *incertae sedis* that were included in this analysis do not constitute a monophyletic group thereby not supporting an expanded concept of Rangomaramidae (Amorim & Rindal, 2007) but further studies with more taxa included are much needed.

##  Supplemental Information

10.7717/peerj.2563/supp-1 Figure S1 Bayesian hypothesis for relationships among selected taxa of Bibionomorpha excluding theSciaroidea *incertae sedis* genera based on DNA sequence data (18S, 28S, CAD, 12S, 16S, and COI) Support numbers refer to posterior probability over 0.95/ bootstrap value over 50/ jackknife value over 50. The branches marked as “//” have been shortened to its half to fit them into the graphic.

10.7717/peerj.2563/supp-2 Figure S2 Maximum likelihood phylogeny of Bibionomorpha based on DNA sequence data (18S, 28S, CAD, 12S, 16S, and COI) Support numbers refer to bootstrap value over 50. The branches marked as “//” have been shortened to its half to fit them into the graphic.

10.7717/peerj.2563/supp-3 Table S1 List of species included in the phylogenetic analysis

10.7717/peerj.2563/supp-4 Table S2 Primers used in this study for PCR amplification and sequencing of the nuclear 18S,28S, and CAD and the mitochondrial 12S, 16S, and COI genes

## Additional Information and Declarations

### Field Study Permissions

The following information was supplied relating to field study approvals (i.e., approving body and any reference numbers): Most of the specimens were collected in localities where no field study permission is required. No taxon included in this study is specially protected by law. For the field research in Central Slovakia permission was issued by the Administration of Muránska planina National Park (No. 2011/00619-Ko and OU-BB-OSZP1-2014/13611-Ku). A part of the material examined was collected within the “Thailand Inventory Group for Entomological Research (TIGER) project” supported by the National Research Council of Thailand and the Department of National Parks, Wildlife and Plant Conservation, Thailand, who gave permission for research and the collection of specimens (see http://sharkeylab.org/tiger/). Two specimens used in the analyses were collected in Kuala Belalong Field Studies Centre, Brunei, in cooperation with the Institute for Biodiversity and Environmental Research, Universiti Brunei Darussalam, who also provided relevant permission (No. UBD-AVC-RI/1.24.1 and BioRIC/HOB/TAD/51-40).

### DNA Deposition

The following information was supplied regarding the deposition of DNA sequences: Sequences have been submitted to the GenBank database under the accession numbers listed in Table 1. Edited sequence alignments used for phylogenetic analyses are available at https://dx.doi.org/10.6084/m9.figshare.c.3458082.v1.

### Data Availability

The following information was supplied regarding data availability: Kaspřák, David (2016): Bibionomorpha alignments. Figshare:
https://dx.doi.org/10.6084/m9.figshare.c.3458082.v1.
