# Supplementary figures and images for: Molecular phylogeny of the megadiverse insect infraorder Bibionomorpha sensu lato (Diptera)
Source: PeerJ. 2016 Oct 18;4:e2563. doi: 10.7717/peerj.2563 (PMC5075709; doi:10.7717/peerj.2563)

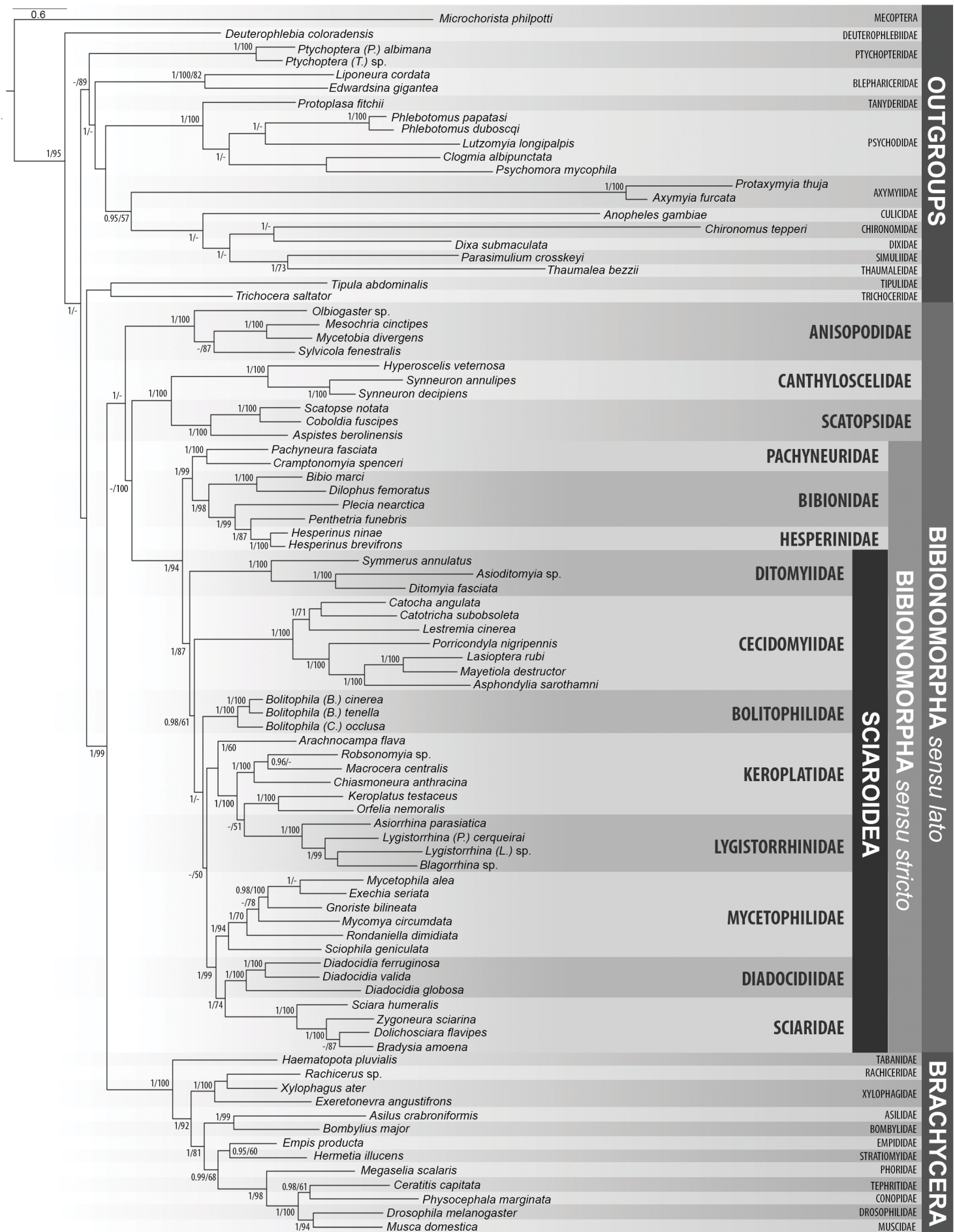

Supplement: Figure S1 — Support numbers refer to posterior probability over 0.95/ bootstrap value over 50/ jackknife value over 50. The branches marked as “//” have been shortened to its half to fit them into the graphic. [file peerj-04-2563-s001.pdf]

# OUTGROUPS

# BIBIONOMORPHA sensu lato

# BRACHYCERA

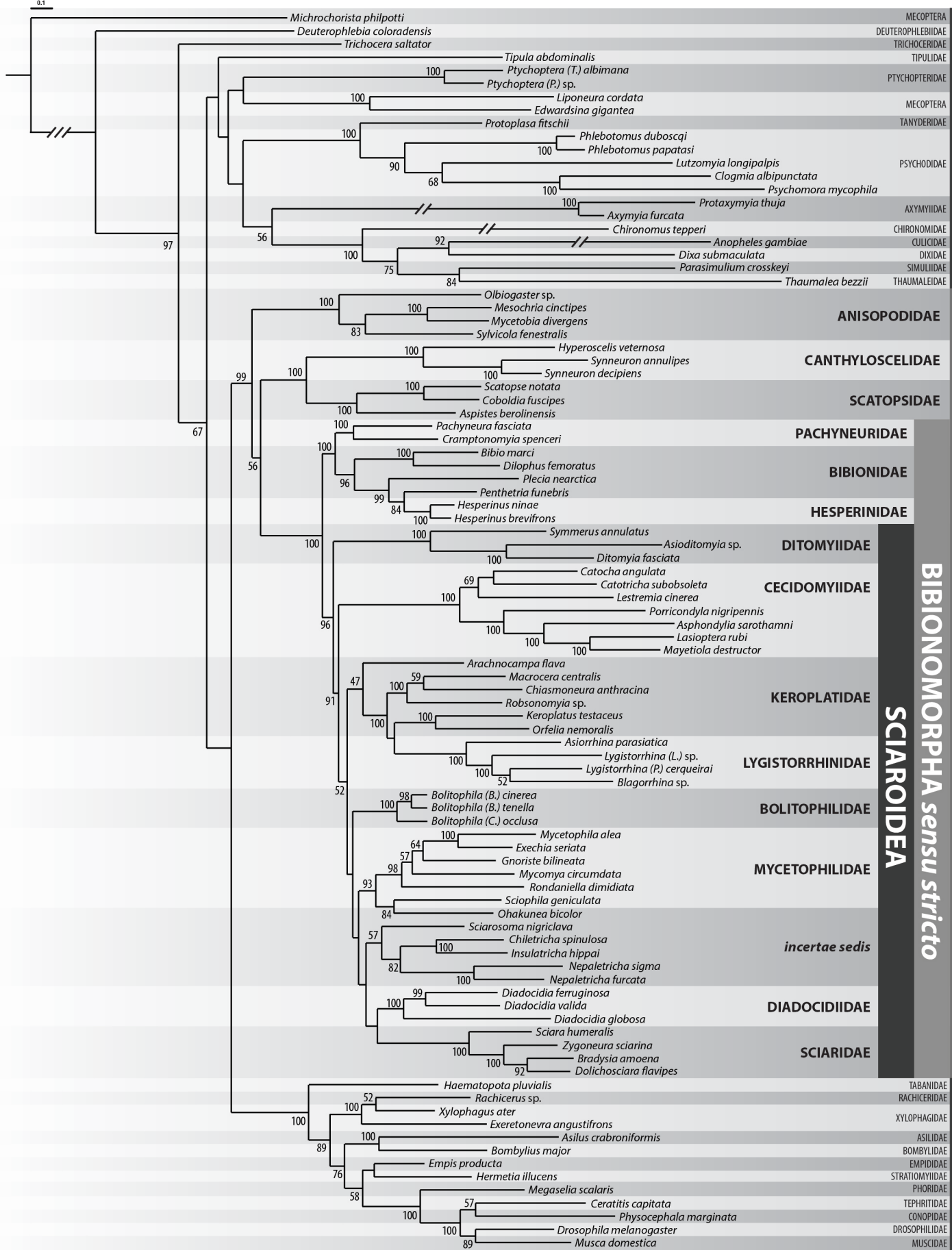

Supplement: Figure S2 — Support numbers refer to bootstrap value over 50. The branches marked as “//” have been shortened to its half to fit them into the graphic. [file peerj-04-2563-s002.pdf]
