# Supplementary material for: Molecular phylogeny of the megadiverse insect infraorder Bibionomorpha sensu lato (Diptera)
Source: PeerJ. 2016 Oct 18;4:e2563. doi: 10.7717/peerj.2563 (PMC5075709; doi:10.7717/peerj.2563)
Supplement: Table S1 [file peerj-04-2563-s003.docx]

Ševčík, J., Kaspřák, D., Mantič, M., Fitzgerald, S., Ševčíková, T., Tóthová, A. & Jaschhof, M. (2016) Molecular phylogeny of the megadiverse insect infraorder Bibionomorpha sensu lato (Diptera).

**Table S1:** List of species included in the phylogenetic analysis. All the specimens are males (if not stated otherwise) and were identified by the authors.

| **Species** | **Authors** | **Sampling locality and year** | **Collection** |
| --- | --- | --- | --- |
| *Asioditomyia* sp. | *unidentified to species* | Brunei, 2014 | JSL-UOC |
| *Asiorrhina parasiatica* | Blagoderov, Hippa & Ševčík 2009 | Thailand, 2008 | JSL-UOC |
| *Asphondylia sarothamni* (female) | (Loew, 1850) | Czech Republic, 2014 | JSL-UOC |
| *Aspistes berolinensis* | Meigen, 1818 | Czech Republic, 2013 | SMOC |
| *Bibio marci* | (Linnaeus, 1758) | Czech Republic, 2013 | JSL-UOC |
| *Blagorrhina* sp. | *unidentified to species* | Malaysia, 2013 | JSL-UOC |
| *Bolitophila* (*Bolitophila* s. str.) *cinerea* | Meigen, 1818 | Slovakia, 2012 | JSL-UOC |
| *Bolitophila* (*Bolitophila* s. str.) *tenella* | Winnertz, 1863 | Czech Republic, 2013 | JSL-UOC |
| *Bolitophila* (*Cliopisa*) *occlusa* | Edwards, 1913 | Slovakia, 2012 | JSL-UOC |
| *Bradysia distincta* | (Stæger, 1840) | Slovakia, 2013 | JSL-UOC |
| *Catocha angulata* | Jaschhof, 2009 | Slovakia, 2014 | JSL-UOC |
| *Catotricha subobsoleta* | (Alexander, 1924) | USA, 2014 | JSL-UOC |
| *Chiasmoneura anthracina* | Meijere, 1913 | Thailand, 2009 | JSL-UOC |
| *Chiletricha spinulosa* | Chandler, 2002 | Chile, 2000 | JSL-UOC |
| *Clogmia albipunctata* | (Williston, 1893) | Czech Republic, 2014 | JSL-UOC |
| *Coboldia fuscipes* | (Meigen, 1830) | Czech Republic, 2011 | SMOC |
| *Diadocidia* (*Adidocidia*) *valida* | Mik, 1874 | Slovakia, 2010 | JSL-UOC |
| *Diadocidia* (*Diadocidia*) *ferruginosa* | (Meigen, 1830) | Slovakia, 2010 | JSL-UOC |
| *Diadocidia* (*Taidocidia*) *globosa* | Papp & Ševčík, 2005 | Thailand, 2008 | JSL-UOC |
| *Dilophus femoratus* | Meigen, 1804 | Slovakia, 2014 | JSL-UOC |
| *Ditomyia fasciata* | (Meigen, 1818) | Czech Republic, 2010 | JSL-UOC |
| *Dixa submaculata*  *Dolichosciara flavipes* | Edwards, 1920  (Meigen, 1804) | Czech Republic, 2015  Slovakia, 2012 | JSL-UOC  JSL-UOC |
| *Exechia seriata* | (Meigen, 1830) | Slovakia, 2012 | JSL-UOC |
| *Hesperinus brevifrons* | Walker, 1848 | USA, 2014 | JSL-UOC |
| *Hesperinus ninae* | Papp & Krivosheina, 2010 | Georgia, 2014 | JSL-UOC |
| *Hyperoscelis veternosa* | Mamaev & Krivosheina, 1969 | Slovakia, 2014 | JSL-UOC |
| *Insulatricha hippai* | Jaschhof, 2004 | New Zealand, 2002 | JSL-UOC |
| *Keroplatus testaceus* | Dalman, 1818 | Slovakia, 2012 | JSL-UOC |
| *Lasioptera rubi* | Loew, 1850 | Czech Republic, 2014 | JSL-UOC |
| *Lestremia cinerea* | Macquart, 1826 | Slovakia, 2012 | JSL-UOC |
| *Liponeura cordata*  *Lygistorrhina cerqueirai*  *Lygistorrhina* sp. | Vimmer, 1916  Lane, 1958  *unidentified to species* | Slovakia, 2014  French Guyana, 2007  Brunei, 2015 | JSL-UOC  JSL-UOC  JSL-UOC |
| *Macrocera centralis* | Meigen, 1818 | Slovakia, 2013 | JSL-UOC |
| *Matileola thaii* | Papp, 2005 | Thailand, 2006 | JSL-UOC |
| *Mesochria cinctipes* (female) | de Meijere, 1913 | Brunei, 2014 | JSL-UOC |
| *Mycetobia divergens* | Walker, 1856 | USA, 2014 | JSL-UOC |
| *Mycetophila alea* | Laffoon, 1965 | Slovakia, 2012 | JSL-UOC |
| *Mycomya circumdata* | (Staeger, 1840) | Slovakia, 2012 | JSL-UOC |
| *Nepaletricha sigma* | Hippa & Ševčík, 2014 | India, 2012 | JSL-UOC |
| *Nepaletricha* *furcata* (female) | Hippa, Chandler & Papp, 2009 | Thailand, 2008 | JSL-UOC |
| *Ohakunea bicolor* | Edwards, 1927 | New Zealand, 2002 | JSL-UOC |
| *Olbiogaster* sp. (female) | *unidentified to species* | Peru, 2010 | JSL-UOC |
| *Orfelia nemoralis* | (Meigen, 1818) | Czech Republic, 2013 | JSL-UOC |
| *Pachyneura fasciata* | Zetterstedt, 1838 | Finland, 2012 | JSL-UOC |
| *Penthetria funebris* | Meigen, 1804 | Slovakia, 2014 | JSL-UOC |
| *Plecia nearctica* | Hardy, 1940 | USA, 2013 | JSL-UOC |
| *Porricondyla nigripennis* | (Meigen, 1830) | Slovakia, 2014 | JSL-UOC |
| *Protaxymyia thuja* | Fitzgerald & Wood, 2014 | USA, 2014 | JSL-UOC |
| *Psychomora mycophila* | (Vaillant, 1988) | Czech Republic, 2014 | JSL-UOC |
| *Ptychoptera albimana*  *Rachicerus* sp. | (Fabricius 1787)  *unidentified to species* | Czech Republic, 2015  Brunei, 2014 | JSL-UOC  JSL-UOC |
| *Robsonomyia* sp. (female) | *unidentified to species* | USA, 2013 | JSL-UOC |
| *Rondaniella dimidiata* | (Meigen, 1804) | Slovakia, 2014 | JSL-UOC |
| *Rutylapa ruficornis* | (Zetterstedt, 1851) | Turkey, 2011 | JSL-UOC |
| *Scatopse notate* | (Linnaeus, 1758) | Czech Republic, 2011 | SMOC |
| *Sciarosoma nigriclava* | (Strobl, 1898) | Finland, 2014 | JSL-UOC |
| *Sciophila geniculata* | Zetterstedt, 1838 | Slovakia, 2013 | JSL-UOC |
| *Symmerus annulatus* | (Meigen, 1830) | Slovakia, 2012 | JSL-UOC |
| *Synneuron annulipes*  *Xylophagus ater* | Lundström, 1910  Meigen, 1804 | Slovakia, 2006  Slovakia, 2014 | JSL-UOC  JSL-UOC |
| *Zygoneura sciarina* | Meigen, 1830 | Slovakia, 2014 | JSL-UOC |

*Abbreviations of the collections*:

JSL-UOC – Jan Ševčík Lab, University of Ostrava, Czech Republic

SMOC – coll. Silesian Museum, Opava, Czech Republic.
