# Supplementary material for: Molecular phylogeny of the megadiverse insect infraorder Bibionomorpha sensu lato (Diptera)
Source: PeerJ. 2016 Oct 18;4:e2563. doi: 10.7717/peerj.2563 (PMC5075709; doi:10.7717/peerj.2563)
Supplement: Table S2 [file peerj-04-2563-s004.docx]

Ševčík, J., Kaspřák, D., Mantič, M., Fitzgerald, S., Ševčíková, T., Tóthová, A. & Jaschhof, M. (2016) Molecular phylogeny of the megadiverse insect infraorder Bibionomorpha sensu lato (Diptera).

**Table S2:** Primers used in this study for PCR amplification and sequencing of the nuclear 18S, 28S and CAD, and mitochondrial 12S, 16S, and COI genes.

| **Gene fragment** | **Primer sequences (5’→3’)** | **Source** |
| --- | --- | --- |
| 12S | CTGGGATTAGATACCCTGTTAT | Cook *et al*. 2004 |
|  | CAGAGAGTGACGGGCGATTTGT | Cook *et al*. 2004 |
| 16S | TAATCCAACATCGAGGTC | Roháček *et al*. 2009 |
|  | CGAAGGTAGCATAATCAGTAG | Roháček *et al*. 2009 |
| 18S | AACCTGGTTGATCCTGCCAGT | Katana *et al*. 2001 |
|  | TGATCCTTCTGCAGGTTCACCTACG | Katana *et al*. 2001 |
|  | AGATACCGCCCTAGTTCTAACC | Campbell *et al*. 1995 |
|  | GGTTAGAACTAGGGCGGTATCT | Campbell *et al*. 1995 |
| 28S | AGAGAGAGAGTTCAAGAGTACGTG | Belshaw *et al*. 2001 |
|  | TAGTTCACCATCTTTCGGGTC | Belshaw *et al*. 2001 |
| COI | GGTCAACAAATCATAAAGATATTGG | Folmer *et al*. 1994 |
|  | TAAACTTCAGGGTGACCAAAAAATCA | Folmer *et al*. 1994 |
|  | AAAATAGGGTCTCCTCCTCC | Ševčík lab |
| CAD | GGNGTNACNACNGCNTGYTTYGARCC | Moulton & Wiegmann 2004 |
|  | TTNGGNAGYTGNCCNCCCAT | Moulton & Wiegmann 2004 |
|  | ACNGAYTAYGAYATGTGYGA | Moulton & Wiegmann 2004 |
|  | TCRTTNTTYTTWGCRATYAAYTGCAT | Moulton & Wiegmann 2004 |

Additional reference:

Folmer, O., Black, M., Hoen, W., Lutz, W., Vrijenhoek, R. (1994). DNA primers for amplification of mitochondrial cytochrome c oxidase subunit I from diverse metazoan invertebrates. *Molecular Marine Biology and Biotechnology*, 3, 294–299.
